# Myocarditis and Subclinical-Like Infection Associated With SARS-CoV-2 in Two Cats Living in the Same Household in France: A Case Report With Literature Review

**DOI:** 10.3389/fvets.2021.748869

**Published:** 2021-10-21

**Authors:** Valérie Chetboul, Pierre Foulex, Kahina Kartout, Anne Marie Klein, Corinne Sailleau, Marine Dumarest, Manon Delaplace, Meriadeg Ar Gouilh, Jeremy Mortier, Sophie Le Poder

**Affiliations:** ^1^École Nationale Vétérinaire d'Alfort, CHUVA, Unité de Cardiologie d'Alfort (UCA), Maisons-Alfort, France; ^2^Université Paris Est Créteil, INSERM, IMRB, Créteil, France; ^3^Clinique Vétérinaire, Paris, France; ^4^École Nationale Vétérinaire d'Alfort, UMR VIROLOGIE, INRAE, ANSES, Laboratoire de santé animale, Université Paris-Est, Maisons-Alfort, France; ^5^Groupe de Recherche sur l'Adaptation Microbienne (GRAM 2.0), Normandie Université, UNICAEN, 13 UNIROUEN, Caen, France; ^6^École Nationale Vétérinaire d'Alfort, CHUVA, Service d'Imagerie Médicale, Maisons-Alfort, France

**Keywords:** cat, COVID-19, echocardiography, heart failure, myocarditis, SARS-CoV-2, troponin-I

## Abstract

This report provides the first clinical, radiographic, echocardiographic, and biological description of SARS-CoV-2-associated myocarditis with a 6-month follow-up in a 5-year-old obese male domestic shorthair cat (Cat-1) presented for refractory congestive heart failure, with high cardiac troponin-I level (5.24 ng/ml), and a large lingual ulcer. The animal was SARS-CoV-2 positive on serology. The other cat living in the same household (Cat-2) never showed any clinical sign but was also confirmed SARS-CoV-2 positive on serology. Both cats were SARS-CoV-2 PCR negative. Cat-1 had closer contact than Cat-2 with their owner, who had been in close contact with a coworker tested PCR positive for COVID-19 (Alpha (B.1.1.7) variant) 4 weeks before Cat-1's first episode of congestive heart failure. A focused point-of-care echocardiography at presentation revealed for Cat-1 numerous B-lines, pleural effusion, severe left atrial dilation and dysfunction, and hypertrophic cardiomyopathy phenotype associated with focal pulmonary consolidations. Both myocarditis and pneumonia were suspected, leading to the prescription of cardiac medications and antibiotics. One month later, Cat-1 recovered, with normalization of left atrial size and function, and radiographic and echocardiography disappearance of heart failure signs and pulmonary lesions. An extensive literature review of SARS-CoV-2-related cardiac injury in pets in comparison with human pathology is discussed.

## Introduction

Since December 2019, a new coronavirus, the severe acute respiratory syndrome–related coronavirus 2 (SARS-CoV-2), has emerged in the human population, leading to an unprecedented pandemic. In February 2020, the first description of SARS-CoV-2 infection of a dog was provided by the authority from Hong Kong (1). This dog was living with a SARS-CoV-2-infected owner and remained asymptomatic. In March 2020, the first clinical case of SARS-CoV-2 infection in a cat, living in Belgium, was reported, with the expression of digestive and respiratory symptoms (2). Since then, numerous infections have been reported in cats and dogs living in households with COVID-19-infected owners (3–8). Recently, infection of pets with the Alpha SARS-CoV-2 variant (previously named B1.1.7) has been described in the USA, Spain, and the UK (9–11). Although the majority of SARS-CoV-2-infected domestic pets remain asymptomatic, few of them may develop digestive, ocular, respiratory, and cardiac signs (3, 10, 12, 13). Interestingly, COVID-19 is also known to be associated with cardiac injury and dysfunction in human patients (14–21).

In the present report, we describe for the first time the clinical, radiographic, echocardiographic, and biological features and follow-up of a SARS-CoV-2-related myocarditis diagnosed in a domestic cat presented for refractory congestive heart failure, living with a SARS-CoV-2-positive asymptomatic cat mate.

Furthermore, an extensive literature review of both SARS-CoV-2-related cardiac injury in human patients and in pets is conducted.

## Case Report Description

A 5-year-old obese (8.5 kg; body condition score of 8/9) neutered male domestic shorthair cat (Cat-1) was referred to the Alfort Cardiology Unit at the Veterinary Hospital of the Alfort National Veterinary School in April 2021 for refractory congestive heart failure (CHF) and general health status worsening over the past 4 weeks, associated with high cardiac troponin-I level (cTnI = 5.24 ng/ml; reference range <0.06 ng/ml). The cat was receiving oral furosemide (1.2 mg/kg q8h), pimobendan (0.07 mg/kg q12h), and clopidogrel (2.2 mg/kg q24h).

Cat-1 was living in a strictly indoor life environment with another domestic shorthair cat (Cat-2; 6.9 kg, body condition score of 6/9). Cat-1 had closer contact than Cat-2 with their owner (e.g., sharing her bed, unlike Cat-2), who had been exposed to a COVID-19-positive and ill coworker [Alpha (B.1.1.7) variant] the day before her coworker's clinical signs onset and 4 weeks before Cat-1's clinical signs occurrence, i.e., acute onset of lethargy, inappetence, and tachypnea/dyspnea. The owner had a negative reverse transcription-polymerase chain reaction (RT-qPCR) nasal test for COVID-19 4 days after close contact with her coworker.

**Figure 1:**
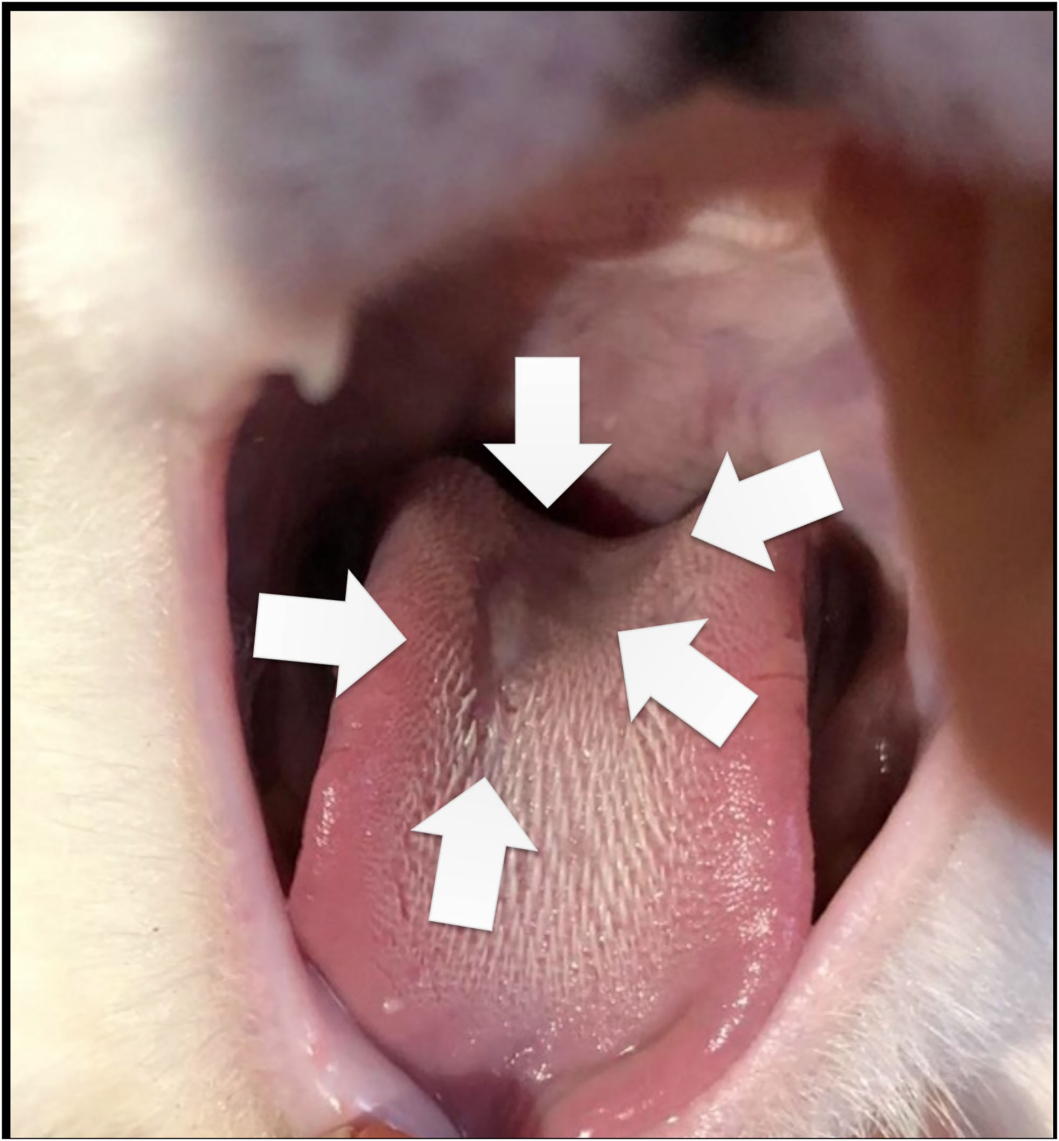
Large ulcer (arrows) observed at the tongue base of Cat-1 at day 0.

At admission, Cat-1 was lethargic. Physical examination at presentation revealed marked dyspnea with tachypnea (respiratory rate = 80 breaths per minute) and paradoxical breathing, associated with a gallop sound, a grade 1/6 left apical systolic heart murmur, and irregular cardiac rhythm. A large ulcer was also found at the tongue base (Figure 1).

Flow-by oxygen therapy and an intramuscular injection of furosemide (2 mg/kg) were implemented. A six-lead ECG was then performed, showing some isolated left premature ventricular complexes with a mean heart rate of 190 bpm. Because the cat was clinically unstable, a focused point-of-care echocardiographic examination was performed by a boarded-certified cardiologist (V.C., Dipl. ECVIM-CA Cardiology), as recommended by the American College of Veterinary Internal Medicine (ACVIM) consensus guidelines (22, 23). To minimize stress during the examination, the cat was gently restrained in standing position, as previously validated by our group and also recommended by the ACVIM consensus guidelines (22, 24).

**Figure 2:**
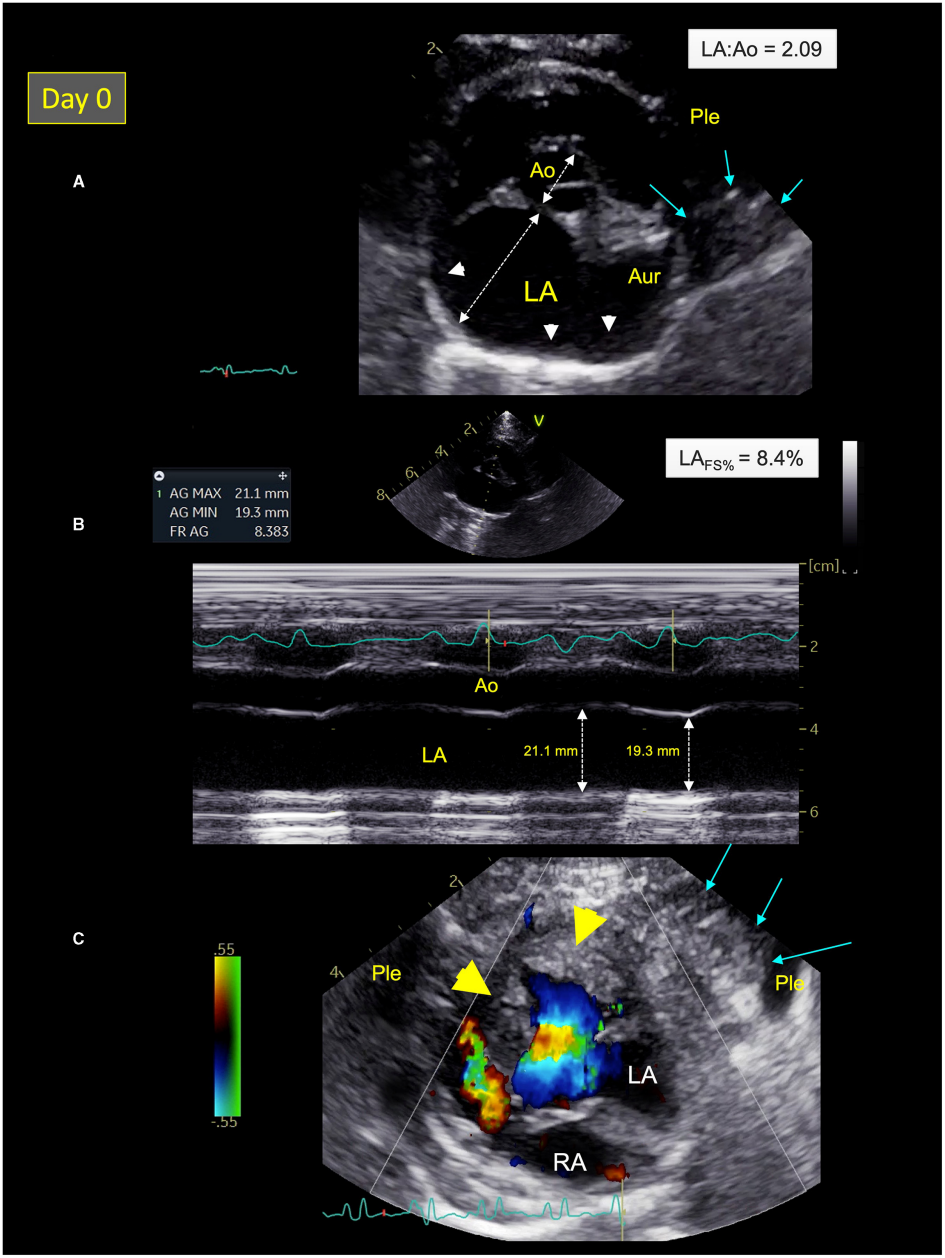
Two-dimensional (2D) right parasternal short-axis view **(A)**, M-mode echocardiogram **(B)**, and color-flow Doppler mode **(C)** from Cat-1 at presentation (day 0). **(A)** This 2D right parasternal short-axis view taken at end-diastole at the level of the aortic valve shows marked dilation of the left atrium (LA) and the left auricle (Aur) with an increased LA:aorta ratio (LA:Ao = 2.09), spontaneous echo contrast (white arrow heads), associated with pleural effusion (Ple), and a pulmonary consolidation near the left auricle (blue arrows). **(B)** This M-mode image obtained from the 2D right parasternal short-axis view taken at the level of the aortic valve confirms a markedly decreased LA fractional shortening (LA_FS%_ = 8.4%). **(C)** This 2D left apical 5-chamber view taken at end-systole shows left ventricular hypertrophy (yellow arrow heads) and a pulmonary consolidation near the left ventricle (blue arrows) surrounded by Ple. RA, right atrium.

The focused point-of-care ultrasound examination revealed numerous B-lines suggestive of pulmonary alveolar disease (e.g., pulmonary edema), mild pleural effusion, and severe left atrial dilation as confirmed by high values of end-diastolic left atrium-to-aorta ratio [LA:Ao = 2.09; reference range ≤ 1.2 (25); Figure 2A], maximum LA diameter on right parasternal 4-chamber view [Max LAD = 22.0 mm; reference range <16.0 mm (26)], and end-systolic LA volume (LAV_s_) assessed by the modified Simpson method of discs from the right parasternal 4-chamber view [6.10 mL; reference range <1.96 mL/cat (27)]. The LA fractional shortening (LA_FS%_) was dramatically decreased [8.4%; reference range >17.5% (28); Figure 2B] confirming marked alteration of LA function associated with blood stasis as shown by decreased LA appendage velocities [maximal velocity = 0.2 m/s; reference range 0.24–1.00 (29)], and spontaneous echo-contrast within the LA and the left auricle.

A hypertrophic cardiomyopathy (HCM) phenotype was also observed, with an irregular thickened pericardium (up to 3.0 mm), and several focal pulmonary consolidations nearby the LA and the left ventricle (LV; Figure 2C). Lastly, important amount of fat was found within both pericardial and pleural spaces.

**Figure 3:**
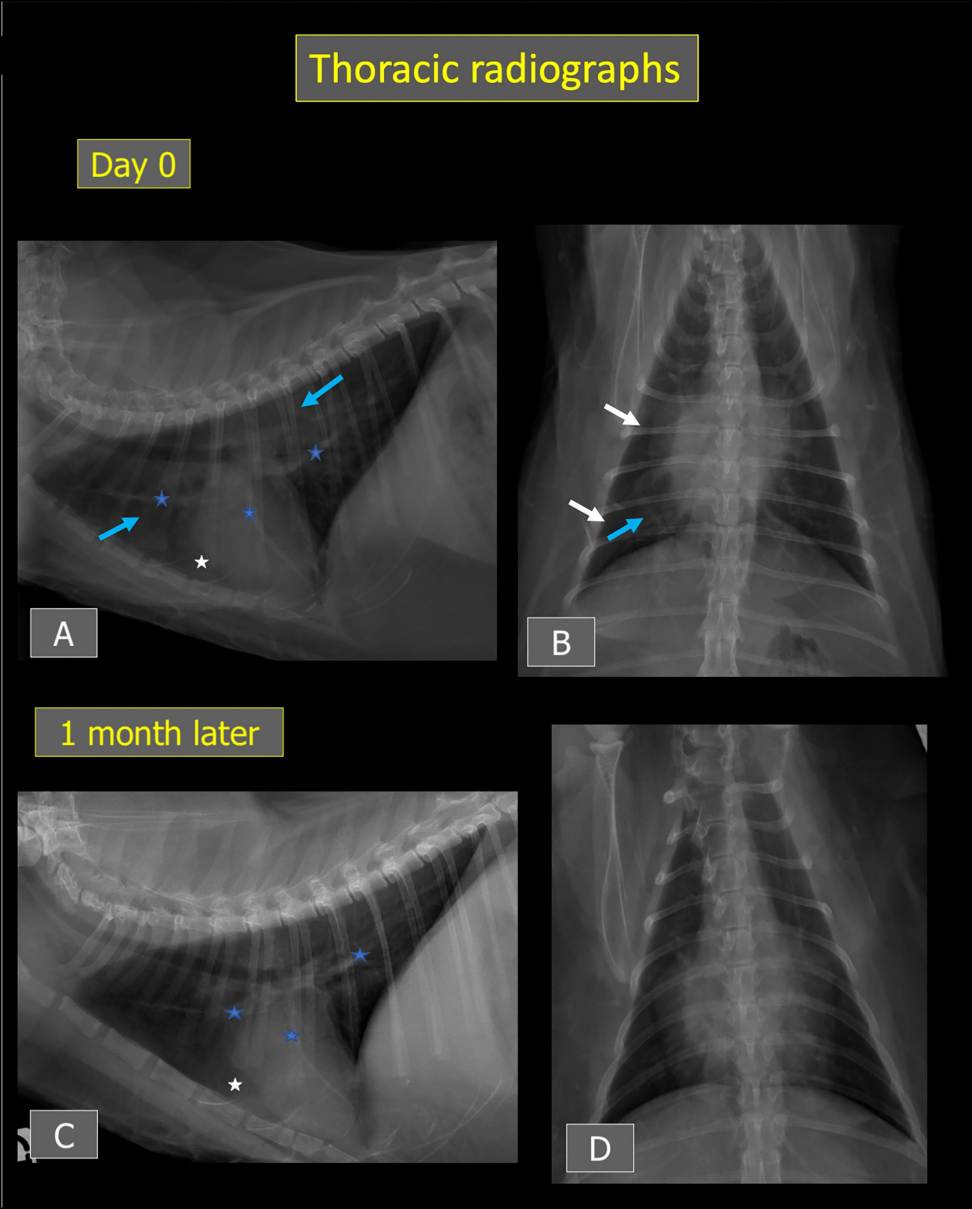
Thoracic radiographs from Cat-1 at presentation **(A,B)** and after 1 month **(C,D)**. An ill-defined unstructured interstitial lung pattern (blue arrows) and thin interlobar fissures (white arrows) consistent with mild pleural effusion are present at presentation. Mild cardiomegaly and subjectively enlarged pulmonary vessels are also noted (blue stars). After 1 month, the pulmonary pattern and interlobar fissures have resolved, and the cardiac silhouette and pulmonary vessels have decreased in size. On **(A,C)**, a large amount of fat is visible around the apex of the cardiac silhouette (white stars).

Thoracic radiographs performed and reviewed by a board-certified radiologist (J.M., Dipl. ECVDI) showed borderline cardiomegaly (vertebral heart score of 8) with subjectively enlarged pulmonary vessels, thin interlobar fissure lines indicative of mild pleural effusion, and a generalized yet inhomogeneous unstructured interstitial lung pattern that was slightly more severe in the right caudal lung lobe. A large quantity of sternal and/or pericardial fat was also present, in agreement with the ultrasound examination (Figures 3A,B).

Considering history, clinical, radiographic, and echocardiographic findings, ACVIM stage C (i.e., decompensated) myocarditis associated with pneumonia was suspected. The cat was discharged with pimobendan and clopidogrel at initial dosages. Oral furosemide was switched for subcutaneous furosemide injections (1.2 mg/kg q8h) and food was supplemented with taurine (700 mg q12h). In addition, dual antibiotic therapy was prescribed: oral clindamycin (9.4 mg/kg q24h) and subcutaneous marbofloxacin injection (2 mg/kg q24h). Extensive investigations were performed, including complete blood count, serum amyloid A protein, serum biochemistry, electrolytes, plasma N-terminal pro-B-type natriuretic peptide (NT-proBNP), and infectious disease testing, i.e., serology for *Toxoplasma gondii, Ehrlichia canis, Anaplasma phagocytophilum, Borrelia burgdorferi*, and *Dirofilaria immitis* (IDEXX SNAP 4Dx), feline leukemia virus (FeLV) p27 antigen and feline immunodeficiency virus (FIV) antibodies (IDEXX laboratory), 16S rRNA PCR test and PCR blood test for *Bartonella henselae*, and calicivirus PCR test (oropharyngeal swab). Lastly, blood sample and nasal, oropharyngeal, and rectal swabs were also collected for SARS-CoV-2 testing, as previously described by our group (4). Serological analyses were performed with two methodologies. Cats' sera were tested with the ID Screen ELISA, SARS-CoV-2 Double Antigen Multi-species) (IDvet, Grabels, France) according to the manufacturer's instructions. The kit specifically detects anti-SARS-CoV-2 nucleocapsid antibodies. The sera were also tested by seroneutralization. Briefly, after heat inactivated treatment, at 56°C for 1 h, serial twofold dilutions of the sera were added to SARS-CoV-2 virus (strain *BetaCoV/France/IDF/200107/2020*, kindly provided by Dr. J.C. Manuguerra, CIBU, Pasteur Institute) at a 50% tissue culture infective dose (TCID50) of 100. After incubation at 37°C for 1 h, the virus serum dilution was inoculated onto Vero E6 cells for 1 h at 37°C. Then, cells were incubated for 3 days. The neutralization titers were determined by the highest dilution that prevented cytopathic effect. In addition, nasal, oropharyngeal, and rectal swabs were submitted to RT-qPCR assays. Briefly, RNA extracts were submitted to standardized one-step RT-PCR 3-Allplex 2019-nCoV assay (Seegene, CE IVD) used as routine diagnosis for SARS-CoV-2 in the virology unit of the University Hospital of Caen, using the manufacturer's instructions, CFX-96 RT System C1000 Thermal Cycler (Biorad) and SARS-CoV2 Viewer for Real-Time instrument version 3.19.003.010 (Seegene). This multiplex system simultaneously targets Orf1, S, and N genes.

All tests were unremarkable, except for high NT-proBNP plasma level (527 pmol/L; reference range <100 pmol/L) and a SARS-CoV-2 serology positive by ELISA (OD value: 1.425; % positivity: 426%). Seroneutralization assay confirmed the presence of specific SARS-CoV-2 antibodies with a serological titer of 1:640. Nasal, oropharyngeal, and rectal swabs were SARS-CoV-2 PCR negative.

Given these results, the other cat living in the same household (Cat-2) was also sampled for the same SARS-CoV-2 tests as well as for NT-proBNP and cTnI levels. Like Cat-1, Cat-2 was found SARS-CoV-2 positive by ELISA (OD value: 0.636; % positivity: 202%) and seroneutralization (titer 1:20), but all collected swabs were RT-qPCR negative. However, NT-proBNP and cTnI levels remained within reference ranges (<24 pmol/L and 0.02 ng/ml, respectively).

**Figure 4:**
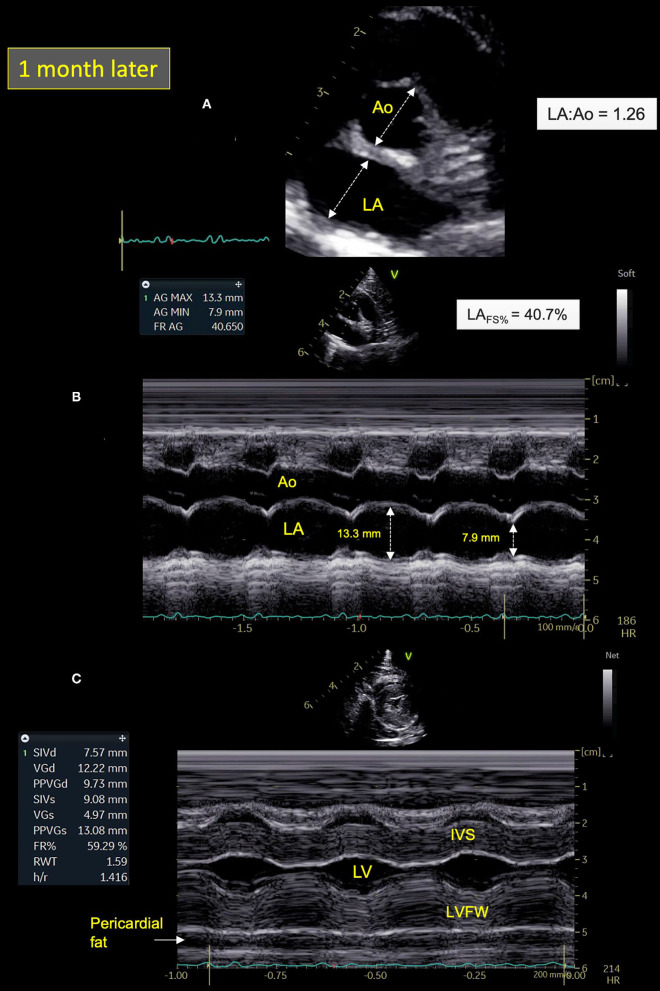
Two-dimensional (2D) right parasternal short-axis view **(A)** and M-mode echocardiograms **(B,C)** from Cat-1 1 month after initial presentation. **(A)** As compared with day 0 (see Figure 2A), this 2D right parasternal short-axis view taken at end-diastole at the level of the aortic valve shows a markedly decreased left atrial size (left atrium:aorta ratio (LA:Ao) = 1.26 vs. 2.09) associated with disappearance of spontaneous echo contrast, pleural effusion, and pulmonary consolidation. **(B)** This M-mode image obtained from the 2D right parasternal short-axis view taken at the level of the aortic valve confirms normalization of the LA fractional shortening (LA_FS%_ = 40.7 vs. 8.4%). **(C)** This M-mode echocardiogram obtained from the right parasternal transventricular short-axis view confirms marked asymmetric left ventricular hypertrophy, more pronounced for the left ventricular free wall (LVFW) than the interventricular septum (IVS), i.e., end-diastolic thicknesses of 9.7 and 7.6 mm, respectively [normal values <6 mm (22)]. Note also posteriorly the important amount of pericardial fat (maximal thickness of 6 mm). LV, left ventricle.

Cat-1's condition gradually improved after treatment adjustment, with no recurrence of respiratory distress at home after 2 weeks and progressive decrease of the size of the lingual ulcer. The cat was re-assessed 1 month later. cTnI level had markedly decreased (0.82 ng/ml). Seroneutralization confirmed the presence of antibodies against SARS-CoV-2 with again a titer of 1:640. Best practice echocardiographic examination with concurrent ECG tracing was performed (22). A dramatic decrease in LA size (LA:Ao = 1.26; max LAD = 12.2 mm; LAVs = 1.50 mL), associated with both normalization of LA function (LA_FS%_ = 40.7%) and Doppler LA appendage maximal velocities (0.73 m/s), was found (Figures 4A,B). Moreover, spontaneous echo-contrast within the LA and the left auricle, pleural effusion, B-lines, and consolidated pulmonary lesions were no longer observed. Similarly, no arrhythmia was detected on the concomitant ECG tracing. An HCM phenotype was confirmed (Figure 4C), associated with impaired LV relaxation, as shown by an inverted maximal early:late diastolic mitral flow velocities ratio [E:A ratio = 0.67; reference range >1 (25, 26)] and decreased mitral annular (E′) velocity [4 cm/s; reference range >6 cm/s (26)], respectively assessed by pulsed-wave Doppler mode and pulsed-wave tissue Doppler imaging, with an E:E′ ratio within upper ranges [11.6; reference range <12 (26)].

Follow-up thoracic radiographs showed a decrease in size of the cardiac silhouette (vertebral heart score of 7.8) and pulmonary vessels with resolution of the pulmonary lesions and pleural effusion (Figures 3C,D).

Owing to the spectacular improvement of LA size and function despite persistent LV hypertrophy, an atrial myocarditis was highly suspected. Hence, the prior medical treatment was continued, with increase of taurine supplementation, addition of fish oil supplementation once a day, and progressive switch of subcutaneous furosemide injections for oral furosemide (1.2 mg/kg q8h).

Six months after onset of clinical signs, Cat-1 is still doing well. Serological analyses were performed again in July 2021 for a follow-up of antibody titers. Cat-1 seroneutralization titer was stable at 1:640 and Cat-2 titer slightly increased to 1:40.

## Discussion

At the end of December 2019, an outbreak of SARS-CoV-2-related pneumonia cases was reported in human patients from Wuhan (Hubei province, China). The corresponding infectious disease, also named COVID-19 (coronavirus disease 19), then rapidly spread all over the world and became a global pandemic declared as public health emergency of international concern by WHO in January 2020 (30). Since then, various human-to-animal SARS-CoV-2 transmissions have been reported, i.e., owner-to-domestic pets [cats and dogs, (1–8, 31)], human-to-wild felids (32, 33), and human-to-mink (34). The susceptibility of several domestic animal species to SARS-CoV-2 infection has also been investigated experimentally, showing that Syrian hamsters, ferrets, and cats are highly permissive to SARS-CoV-2 infection, with possible airborne transmission among these animals (35). Conversely, SARS-CoV-2 replicates poorly in dogs, pigs, cows, and not in chickens and ducks (35). At the time of writing, although human-to-cat transmission has been reported, there is no evidence of transmission in the opposite direction, i.e., cat-to-human transmission (36).

The majority of SARS-CoV-2-infected domestic pets are reported to be subclinical, with few of them showing mild to severe ocular, digestive, and respiratory signs, e.g., conjunctivitis, rhinitis, sneezing, cough, and less commonly respiratory distress (3, 12, 13). The present report provides the first clinical, radiographic, echocardiographic, and biological accurate description, with a 6-month follow-up, of a SARS-CoV-2-related myocarditis in a domestic shorthair cat (Cat-1) referred for refractory CHF, characterized by combined pulmonary edema and pleural effusion. Interestingly, a sudden rise in incidence of myocarditis (i.e., from 1.4 to 12.8%; 8.5% in cats and 4.3% in dogs) has been recorded in a British veterinary Cardiology Department between December 2020 and February 2021 concomitantly to the outbreak of COVID-19 human pandemic in the UK (10). Myocarditis was reported by these authors in six domestics pets (four cats and two dogs) infected by the Alpha (B.1.1.7) variant of SARS-CoV-2, as confirmed by either positive serology or by RT-qPCR assays from rectal swabs (10). In the latter report (10), none of the six affected pets had a previous history of heart disease and their clinical presentation was similar, characterized by acute onset of lethargy, inappetence, tachypnea/dyspnea secondary to CHF with profound impairment of the general health status, and elevated cTnI, as was exactly the case here for Cat-1.

In humans, SARS-CoV-2 is known to affect multiple organ systems, particularly the lungs and heart, with a wide spectrum of presentations including acute myocardial injury reported in up to 38% of hospitalized patients (14–21). SARS-CoV-2-related acute myocardial injury results from various pathophysiologic mechanisms, e.g., coronary microvascular thrombosis, respiratory failure and hypoxemia with secondary ischemic myocardial lesions, and also myocarditis mainly related to hyper-inflammation and cytokine storm mediated through pathologic T cells and monocytes (14, 15, 20, 21). Myocardial SARS-CoV-2 infection, which is common in patients dying from COVID-19 but often with only rare infected cells, can also contribute to a lesser extent to the development of inflammatory cardiac lesions (20, 21). In addition, SARS-CoV-2 has been demonstrated to use the angiotensin I converting enzyme 2 (ACE2) as a cellular entry receptor (37, 38). ACE2 is a peptidase involved in the renin–angiotensin–aldosterone system regulation, expressed in abundance in several tissues, including intestine, heart muscle, kidneys, and lungs (37, 38). Binding of SARS-CoV-2 spike protein to ACE2 leads to viral entry in target cells, but also to renin–angiotensin–aldosterone imbalance, with decreased conversion of angiotensin II to angiotensin (1–7), which may contribute to cardiovascular injury and CHF (37, 38). In the present report, endomyocardial biopsies under general anesthesia could not be performed because Cat-1 was suffering from severe CHF, so the presence of SARS-CoV-2 within the myocardium could not be investigated. Whether SARS-CoV-2 directly induced Cat-1's myocarditis or indirectly, e.g., through a multisystem inflammatory syndrome as described in human patients (14, 15, 20, 21), thus remains unknown.

Interestingly, unlike Cat-2, Cat-1 was an obese cat, with an 8/9 body condition score and important amount of fat within the pericardial and pleural spaces at both echocardiographic and radiographic examinations. In human patients, obesity is a well-known major risk factor for COVID-19 cardio-pulmonary complications and mortality, as is the amount of epicardial adipose tissue (39, 40). Epicardial adipose tissue has been suggested to play a role in COVID-19-related myocarditis (40, 41), as it expresses high levels of ACE2 and as it also represents an inflammatory depot in contiguity with the myocardium, characterized by macrophage infiltrates and proinflammatory cytokines (e.g., interleukin-6). This can lead to myocardial inflammation directly *via* the vasa vasorum or through paracrine pathways (40).

The thoracic radiographs of Cat-1 revealed mild cardiomegaly and enlarged pulmonary vessels, consistent with the echocardiographic diagnosis of myocarditis and left-sided CHF. The pulmonary lesions could be explained either by the presence of cardiogenic pulmonary edema and/or by COVID-19-related pneumonia. A bilateral unstructured interstitial lung pattern has been described in human patients with COVID-19, although consolidations are more frequent (42, 43). The distribution of the pulmonary lesions is variable in COVID-19 pneumonia, and although it is more frequently peripheral, perihilar and diffuse patterns are not uncommon (42, 43). Similarly, mild pleural effusion has been described both in human patients with COVID-19 and in cats with left-sided congestive heart failure; therefore, its origin in our cat is debatable (42–44).

In the present report, a large ulcer was also found on Cat-1's tongue base at the time of presentation, which may have contributed to inappetence. Various oral manifestations of COVID-19 have been reported in human patients, including dry mouth, gustatory impairment, gingivitis, sialadenitis, vesicular and ulcerative mucosal lesions predominantly located on dorsal surface of the tongue (as in our case), hard palate, and labial mucosa (45–47). The exact pathogenesis of such oral signs remains uncertain, probably involving several mechanisms, such as deterioration of the general health status, hypersensitivity of drugs used for COVID-19 treatment, stress, immunosuppression, vasculitis, and hyper-inflammatory response secondary to SARS CoV-2 infection as predisposing factors (45–47). In addition, high expression of ACE2, recognized as the SARS-CoV-2 entry ligand receptor, has been reported in cells of the oral cavity, predominantly in epithelial cells of the tongue, and in buccal and gingival tissues at a lesser extent, thus predisposing to oral SARS-CoV-2 infection with a local virus effect (48).

In the present report, both cats were shown SARS-CoV-2 positive by serology but displayed negative RT-qPCR results for rectal, nasal, and oropharyngeal swabs. Given the clinical manifestation with lung pathology, RT-qPCR on fine-needle lung cytoponction would have been interesting for analysis. However, swab samples were collected quite late after Cat-1's onset of clinical signs (i.e., 1 month after), which may explain the negative RT-qPCR results. In experimental infections, cats shed virus only up to 14 days (49). Serological titers remain identical for Cat-1 during the 3 months of follow-up, thus preventing possible re-infection within a short time. However, long-term follow-up over one year will be necessary to evaluate the duration of immunity.

The two cats were living in the same household and had no outdoor access, thus highly supporting an owner-to-cat SARS-CoV-2 transmission. The asymptomatic owner was suspected of being infected by SARS-CoV-2 because of a close contact with an ill positive coworker (Alpha (B.1.1.7) variant) the day before clinical signs onset of the latter. Nevertheless, the owner was tested RT-qPCR negative for COVID-19 4 days after exposition to her coworker. However, false-negative results of initial RT-qPCR assays for COVID-19 have been reported in 1.8 to 5.8% infected human patients according to studies (50). This case report illustrates that, as recommended by the OiE (World Organisation for Animal Health), people who are suspected or confirmed to be infected with SARS-CoV-2 should avoid close contact with their companion animals (50). Importantly, there is currently no justification in taking measures that may compromise the welfare and health of the latter, as there is no evidence that dogs and cats can infect humans (36, 51).

Cat-1 was more predisposed to develop clinical signs than Cat-2, owing to its body condition score (obesity), associated with the presence of epicardial adipose tissue confirmed by radiographic and ultrasound examination, and a closer contact with the owner (e.g., sharing her bed, unlike Cat-2). As cats have been shown to be susceptible to infection by SARS-CoV-2, which can then replicate efficiently and be transmissible to naïve cats (35), whether Cat-2 was infected by Cat-1 or by the owner remains unknown.

This report presents several limitations. First, thoracocentesis and fine-needle biopsy of the pulmonary lesions detected at presentation (using focused point-of-care ultrasound examination) were not performed for ethical reasons because Cat-1 was clinically unstable. The hypothesis that the virus initially caused focal pneumonia, which then led to cardiac infection, cannot therefore be excluded. Similarly, endomyocardial biopsies could not be performed for the same ethical reasons, and thus the link between SARS-CoV-2 infection and myocarditis remains a speculation for Cat-1. Lastly, primary HCM responsible at least in part for the HCM phenotype cannot also be excluded, as no echocardiographic examination was performed prior to CHF onset.

In conclusion, to the best of the authors' knowledge, the present case is the first clinical, radiographic, echocardiographic, and biological description of SARS-CoV-2-related myocarditis in a domestic cat, over a 6-month period after clinical signs onset. Obesity, epicardial adipose tissue, and close contact to the infected owner were highly probable predisposing factors of Cat-1 to cardiorespiratory failure, as compared with its SARS-CoV-2-positive but asymptomatic cat mate. Moreover, this case report illustrated that SARS-CoV-2 infection should currently be included in the differential diagnosis of feline myocarditis, unstructured interstitial lung pattern on thoracic radiographs, and tongue ulcer. Interestingly, most clinical abnormalities (e.g., obesity, large lingual ulcer, dyspnea) as well as biological and imaging alterations detected on Cat-1 were similar to those reported in human SARS-Cov-2 infection, thus suggesting that small animals could be relevant spontaneous COVID-19 clinical models, potentially improving our knowledge on the human disease. Further prospective studies in large populations of cats and dogs living in households with COVID-19 infected owners are now needed to determine the proportion of animals with high cTnI levels suggestive of myocarditis. Inversely, clinical research including systematic SARS-CoV-2 testing on cats and dogs with presumed myocarditis would be of interest to better assess the potential link between SARS-CoV-2 infection and myocarditis in small animals.

## Data Availability Statement

The original contributions presented in the study are included in the article/supplementary material, further inquiries can be directed to the corresponding author.

## Author Contributions

All authors contributed to the diagnosis, follow-up and interpretation for the 2 presented SARS-CoV-2 cats, regarding clinical and imaging examinations (VC, PF, KK, JM, and AK), virological tests (CS, MDu, MDe, MG, and SL), manuscript revision, read, and approved the submitted version. VC, PF, and KK wrote the first draft of the manuscript. JM and SL wrote sections of the manuscript.

## Funding

This work was supported by the European ICRAD MUSECoV project and by WHO through the SARS-CoV-2 EvoZoone project. The strain *BetaCoV/France/IDF/200107/20* was supplied by the Urgent Response to Biological Threats (CIBU) hosted by Institut Pasteur (Paris, France) and headed by Dr. Jean-Claude Manuguerra. The human sample from which strain *BetaCoV/France/IDF/200107/*2020 was isolated has been provided by Dr. Paccoud from the La Pitié-Salpétrière Hospital. We also sincerely acknowledge the Fondation Un Cœur (Foundation under the aegis of the Fondation de France) and the Vetoquinol company for having sponsored the research assistant position of PF.

## Conflict of Interest

The authors declare that the research was conducted in the absence of any commercial or financial relationships that could be construed as a potential conflict of interest.

## Publisher's Note

All claims expressed in this article are solely those of the authors and do not necessarily represent those of their affiliated organizations, or those of the publisher, the editors and the reviewers. Any product that may be evaluated in this article, or claim that may be made by its manufacturer, is not guaranteed or endorsed by the publisher.
